# Supplementary material for: Chromothripsis during telomere crisis is independent of NHEJ, and consistent with a replicative origin
Source: Genome Res. 2019 May;29(5):737–49. doi: 10.1101/gr.240705.118 (PMC6499312; doi:10.1101/gr.240705.118)
Supplement: Supplemental Material [file supp_gr.240705.118_Supplemental_file_1.zip › contigs/annotated_contigs/DB79/contig.2.DB79_length_482_mean_cov_6.78630705394.docx]

**DB79_length_482_mean_cov_6.78630705394**

```
TTTTGTATTTTTAGTAGAGACAGGGTTTCACTATGTTGGCCGGGTTGGTCTCGAACTCTTGACCTTGTGATCCACCCGCCTCGGCCTCC
 >chr9:123603065-123603218 + E=2e-80 p=0e+00
CAAAGTGCTGGGATTACAGGCGTGAGCCACCGTGCCTGGCCTAACTTTTGTATTTTTAGT|AGAG|TTCAGGGTTGCAGTGAGCTATGA
                                                             >chr9:114293399-114293707
TCATGCCACTGCTGCCTTCCAGCCTGGGCAACAGAGCAAGACCCTGTCTCTAAAAAAAATAGAATAAATGTAATCTCCCTTTCAAGGAA
+ E=7e-171
CTGACGCCTCATTTTCCACATGCAATTTTGTTTCAGTTCACAGAATGCCATGAGTCTGATTGTAATGAAACAAAAACACTTACTAAAAT

TCAGCAGTTACCACTATTAAAATTAGTCAGGTGAAATAGAAGTTCATTTCCTTCTTTTTTTTTCTTTCTTGAGACAGAGTTTTACTCCG

TTGCCCAGGCTGGA|AGATCGGAAGAGCGTCGTGTAGGGA
```
